# Suicide Among Seafarers in Montenegro and the Balkan Region: Epidemiological Data and Occupational Risks

**DOI:** 10.1192/j.eurpsy.2026.12070

**Published:** 2026-07-31

**Authors:** B. Coric, T. Brandmajer, F. Mihailovic, S. Sekularac Ivosevic

**Affiliations:** 1 Department of therapy-resistant psychoes, Special Hospital for Psychiatry Dobrota, Kotor; 2 Psychiatric clinic - psychosis department, Clinical Center of Montenegro, Podgorica; 3 Faculty of Maritime Studies Kotor, University of Montenegro, Kotor, Montenegro

## Abstract

**Introduction:**

Suicide is one of the leading causes of premature mortality worldwide, with over 720,000 deaths annually (WHO, 2025). Seafarers are recognized as a high-risk occupational group due to isolation, long shifts, separation from family, stigma, and limited access to health and psychological services. While research in Scandinavia and the United Kingdom indicates elevated suicide mortality among seafarers (Roberts et al., 2013; Eriksson et al., 2024), systematic data and analyses are lacking in Montenegro and the Balkan region.

**Objectives:**

To analyze national suicide trends in Montenegro for the period 2014–2024, compare them with European averages, and synthesize international evidence on suicide risk among seafarers, with a particular focus on crew members.

**Methods:**

Official data on suicide mortality were collected from the Police Directorate/Ministry of Interior of Montenegro (2014–2024). Crude suicide rates were calculated using national population denominators (\~620,000). Standardized suicide rates for the EU-27 (ICD-10 X60–X84, Y87.0) were obtained from Eurostat. In addition to quantitative analysis, a narrative synthesis of international literature on suicide among seafarers was conducted, including a systematic review (Mathieu et al., 2022), cohort studies (Eriksson et al., 2024; Iversen, 2011), and a review of specific methodological limitations and underreporting issues (Baumler & Carrera-Arce, 2025).

**Results:**

In Montenegro, between 2014 and 2024, the number of suicides ranged from 83 to 126 per year, corresponding to crude rates of 13–20 per 100,000 population. In 2022, 126 suicides were recorded, while in 2024 there were 83 cases. In the EU-27, standardized rates were 10.2/100,000 (2021) and 10.6/100,000 (2022). Although national data do not record occupation, international evidence indicates an increased risk of suicide among seafarers, particularly among crew members. Swedish cohort studies have confirmed elevated standardized mortality ratios (SMRs) for suicide and external causes of death, while systematic reviews and thematic analyses highlight the issue of underreporting and “disappearances at sea” as potentially concealed suicides.

**Conclusions:**

Suicide rates in Montenegro remain high, with fluctuations that often place them above the EU average. Although official statistics currently do not allow for occupational breakdown, international research confirms that seafarers represent a high-risk group. Priorities for the coming period include: (i) improving mortality statistics through the recording of occupation, (ii) mandatory reporting of seafarer deaths in accordance with the MLC 2022 amendments, and (iii) developing preventive programs and mental health support measures for seafarers in Montenegro and the wider Balkan region.

**Disclosure of Interest:**

None Declared

